# Children Who Read Words Accurately Despite Language Impairment: Who Are They and How Do They Do It?

**DOI:** 10.1111/j.1467-8624.2009.01281.x

**Published:** 2009-03

**Authors:** Dorothy V M Bishop, David McDonald, Sarah Bird, Marianna E Hayiou-Thomas

**Affiliations:** University of Oxford; University of York

## Abstract

Some children learn to read accurately despite language impairments (LI). Nine- to 10-year-olds were categorized as having LI only (*n*=35), dyslexia (DX) only (*n*=73), LI + DX (*n*=54), or as typically developing (TD; *n*=176). The LI-only group had mild to moderate deficits in reading comprehension. They were similar to the LI + DX group on most language measures, but rapid serial naming was superior to the LI + DX group and comparable to the TD. For a subset of children seen at 4 and 6 years, early phonological skills were equally poor in those later classified as LI or LI + DX. Poor language need not hinder acquisition of decoding, so long as rapid serial naming is intact; reading comprehension, however, is constrained by LI.

Children with literacy difficulties have informed our understanding of typical reading development. In particular, research on developmental dyslexia has indicated that phonological processing plays an important role in literacy acquisition. Many children with dyslexia find it difficult to identify and manipulate speech sounds, even when the task does not involve any written language (see Snowling, 2000, for a review). Furthermore, these children often make errors repeating polysyllabic nonwords or real words (Snowling, 2001). Such findings support the view that the ability to identify, access, remember, and manipulate phonological representations is crucial for learning to decode written text into oral language.

Developmental dyslexia is diagnosed when a child has difficulty learning to read words accurately and fluently for no apparent reason (Lyon, 2003), but single-word reading is not the only skill needed to be a proficient reader. According to the “simple view of reading” (Hoover & Gough, 1990), to predict a child’s level of reading comprehension, we need to take into account both decoding skill and oral language comprehension. Hoover and Gough claimed that decoding and language comprehension are separable, especially in the early stages of learning to read. Their theory predicts that children with poor oral comprehension will not be proficient readers even if they have excellent decoding skills. In general, although there is some debate as to whether the simple view of reading provides a complete account, research on individual differences in literacy development has found that it explains much of the variance in reading comprehension (e.g., Adlof, Catts, & Little, 2006; Chen & Vellutino, 1997; Johnston & Kirby, 2006; Juel, 1998; Savage, 2006).

Children with developmental language impairment (LI) provide an interesting test case for studying the role of oral language skills in learning to read. LI is diagnosed when a child’s language development lags behind other skills for no apparent reason, despite normal-range nonverbal ability. According to the simple view of reading, reading comprehension will be impaired if either receptive language is inadequate or decoding is poor. Many children with LI have phonological impairments similar to those seen in dyslexia, and in many cases these are accompanied by receptive language difficulties. This means that both components of the “simple view” are affected, and it is not surprising to find that literacy skills in this population are often very poor (Bishop & Snowling, 2004). Nevertheless, Catts, Adlof, Hogan, and Weismer (2005) identified a subset of children with LI who were competent at single-word reading, and they showed that the children did not have phonological impairments. In a similar vein, Kelso, Fletcher, and Lee (2007) identified a subset of children with LI who had good decoding skills, and they showed the children had relatively unimpaired phonological skills. Neither of these studies, however, took children’s reading speed into account. If children with LI who read accurately do so slowly and laboriously, then their literacy attainments could have been overestimated by use of an untimed reading test (see Fuchs, Fuchs, Hosp, & Jenkins, 2001).

Another point to note is that if children with receptive LI do have good decoding skills, then, according to the simple view, they would be expected to have literacy difficulties when assessed by reading comprehension tests. This is exactly what was found in the study by Kelso et al. (2007).

Studies of children with dyslexia have identified a third skill that is related to development of fluent reading, rapid serial naming. Rapid naming of repeating series of familiar pictures, colors, or alphanumeric characters is often poor in children with dyslexia (Denckla & Cutting, 1999). This link is particularly evident in regular orthographies, where most children learn to decode accurately using letter–sound correspondences, but dyslexic readers, who typically are poor at rapid serial naming, are characterized by slow, nonfluent reading (Wimmer, Mayringer, & Landerl, 2000). Rapid serial naming is often regarded as a test of phonological retrieval, but performance on this measure can be dissociated from other phonological processing tasks (e.g., Kirby, Parrila, & Pfeiffer, 2003; Manis, Seidenberg, & Doi, 1999; Wolf et al., 2002), indicating that it is not just an alternative measure of phonological skill. Typically, children with the most severe reading disabilities show deficits in both rapid serial naming and phonological awareness (Morris et al., 1998).

We may distinguish two explanations for the link between dyslexia and slow serial naming. The first maintains that the two go together because rapid naming uses the same brain circuitry as reading. This idea dates back to Geschwind’s (1965) insight that both reading and naming involve making visual–auditory associations; he suggested that dyslexia might involve late maturation of the brain region that mediates such associations, the angular gyrus. More recently, McCrory, Mechelli, Frith, and Price (2005) showed that both reading and picture naming involve lexical retrieval of familiar phonological sequences mediated by the left occipito-temporal region, an area that is underactivated in individuals with dyslexia. Dehaene (2005) proposed that when a child learns to read this region of the brain is redeployed; its normal role is object recognition, but it becomes specialized for recognizing letters and words as well. On this view, we might expect decoding skill to be an indicator that this region is functioning well; if so, rapid serial naming should be unimpaired in children who can decode fluently, regardless of their language status.

An alternative hypothesis regards slow serial naming as a correlate of poor oral language development. Children with LI often give slow responses on confrontation naming tasks (Lahey & Edwards, 1996), and this could reflect generalized slowing and/or poor organization of lexical representations, rather than a specifically phonological problem. If this were the case, we should find deficits on rapid serial naming in most children with LI, regardless of their literacy skills. Furthermore, this line of explanation would predict that rapid serial naming should be impaired only in those dyslexics who had poor oral language skills.

In sum, children who learn to read words accurately despite LI merit more detailed examination, because if we could understand how they master single-word reading, this might give some insights into how to help other children with literacy problems. In most studies, these cases are not distinguished from other children with LI, so we know little about them.

In the current study we aimed to specify the characteristics of children who read words rapidly and accurately despite LI, to consider whether oral language difficulties of these children are qualitatively or quantitatively different from those who have LI in association with dyslexia. In particular, we asked the following questions: (a) Some children read single words rapidly and accurately despite LI; is their reading comprehension poor in relation to reading accuracy? (b) Do these children simply have less severe language difficulties than other children, or is their profile of language skills different? In particular, are phonological skills and/or rapid serial naming intact? (c) Insofar as children with LI but good single word reading do have different language profiles from other children with LI, is this evident before children start to learn to read? This question is important because some language abilities, such as phonological processing and rapid serial naming, might be influenced by literacy skills. Thus comparison of children’s profiles in the preschool years helps determine which cognitive strengths and deficits might be causes rather than consequences of good or poor literacy.

These questions were addressed using data from same-sex twins aged 9–10 years who had been selected by oversampling children at risk of problems with language or literacy (see below for details). For some of these children, results were available from previous waves of data collection at 4 and 6 years of age. Children were categorized according to the 9-year-old test results as having dyslexia (DX), LI, LI + DX, or typical development (TD). Note that a twin sample was used because of our interest in heritability of LI, but no genetic analyses are reported here, as the current focus is on the cognitive profile of children with LI and normal reading skill rather than on etiology of these impairments.

## Method

### Participants

**Figure 1:**
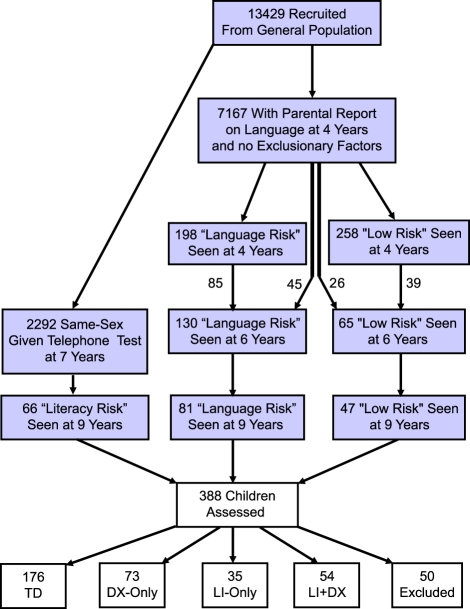
Flow chart showing selection of children for current study in relation to previous waves of data collection from Twins Early Development Study. *Note.* The current sample was selected to be overrepresentative of children with language or literacy problems. Numbers in shaded cells denote twin pairs; numbers in unshaded cells denote individual children.

Same-sex twin pairs came from the Twins Early Development Study (TEDS), a community sample of twins born in England and Wales between 1994 and 1996 (Trouton, Spinath, & Plomin, 2002). In this report we focus on a subsample of same-sex twin pairs who were seen at 9–10 years of age for individual assessment and whose test scores at that age were used as the basis for classifying DX and LI status (see below). These twins were deliberately selected to oversample children with language or literacy problems, using information from earlier waves of data collection to identify those at risk. Figure 1 shows how the current sample was selected and how they relate to previous waves of data collection from TEDS by parental report at 4 years (Colledge et al., 2002), in-home testing at 4 years (Kovas et al., 2005), in-home testing at 6 years (Bishop, Laws, Adams, & Norbury, 2006), and telephone testing at 7 years (Harlaar, Hayiou-Thomas, & Plomin, 2005). Language risk status (“language risk”) was determined on the basis of parental responses to a questionnaire completed when the child was 4 years of age, which allowed us to identify pairs where one or both twins (a) were not talking in full sentences, (b) had expressive vocabulary below the 15th percentile, or where (c) the parent was concerned because the child’s language was developing slowly (see Bishop, Price, Dale, & Plomin, 2003). In the TEDS sample as a whole, 12% of twin pairs met this criterion of language risk at 4 years of age. A subset of these children was seen for individual testing at 4 years of age (Kovas et al., 2005). Because children were distributed across the United Kingdom, selection of cases for testing was determined by availability of the children when testers were in their area. At 6 years of age a further group of TEDS twins was selected such that two thirds of them met criteria for “language risk,” whereas the remaining one third were a low-risk sample (see Bishop et al., 2006). (Around two thirds of these children had also been seen at 4 years.) All available children from that study were seen again for the current study at 9–10 years of age, giving 128 twin pairs after excluding those meeting exclusionary criteria (see below). Note that parental report was not used as the basis for classification in the current study; a child’s preschool risk status was used simply to ensure that we included a high proportion of children who were likely to meet our psychometric criterion of LI at 9 years.

A further 66 twin pairs were selected from the original TEDS cohort to include children at high risk of literacy problems. These children had been assessed by telephone at 7 years of age (Harlaar et al., 2005), when reading ability was measured using the Test of Word Reading Efficiency (TOWRE; Torgesen, Wagner, & Rashotte, 1999). Children at risk of dyslexia were those who scored at least 1.33 *SD* below age level on this measure but scored within 1 *SD* of normal limits on a nonverbal ability composite. Note that the 7-year-old test scores were not analyzed in the current study but were used to select children who were likely to show evidence of dyslexia when seen at 9 years of age.

The numbers shown in Figure 1 are totals after excluding cases where parents reported that one or both twins had sensorineural hearing loss, physical handicap, autism, or another syndrome affecting cognitive development. Twin pairs that included a child who failed a hearing screen when assessed (average hearing threshold for frequencies 500–400 Hz higher than 26 dB in the better ear) or where a child scored above the cutoff for autism on the Autism Screening Questionnaire (Berument, Rutter, Lord, Pickles, & Bailey, 1999) were also excluded, as well as families where English was not the only language spoken in the home. The participants were selected to be White in order to reduce genetic heterogeneity in molecular genetic studies with this sample. After these exclusions (*N*=30 pairs), the sample consisted of 388 children from 194 twin pairs, of whom 76% included at least one twin with language or literacy problems. For two thirds of the cases from “language risk” and “low-risk” samples (78 twin pairs), data were available from earlier waves of testing at both 4 and 6 years of age.

Signed consent for their children’s participation was obtained from parents at each wave of data collection. Ethics approval for data collection at 6 and 9 years of age was obtained from Oxford University’s Experimental Psychology Research Ethics Committee and for the 4-year data collection from the Joint South London and Maudsley and the Institute of Psychiatry NHS Research Ethics Committee.

### Individual Assessment at 9 Years

Because twins were located over a wide geographical range and were often seen at school, the battery was designed to take no longer than 2 hr to administer so that both members of a twin pair could be seen in 1 day. The assessment battery administered at 9 years is shown in Table 1. Tests were selected to assess expressive and receptive language, reading and spelling, and skills related to these domains, including rapid serial naming, nonword repetition, and verbal learning. Psychometric information on published tests was variable, but where estimates of reliability were provided, these are given, and elsewhere the monozygotic (MZ) intraclass correlation is provided from the current sample; this may be taken as a lower bound estimate of reliability. Phonological awareness was not assessed at 9 years, because of concerns that measures that are sensitive in this age group place demands on executive as well as phonological segmentation skills and may be influenced by use of orthographic knowledge (e.g., Castles, Holmes, Neath, & Kinoshita, 2003).

**Table 1:** Psychometric Assessments of Language, Literacy, and Nonverbal Ability at 9 Years

Domain^a^	Instrument	Content	Reliability^b^
	*Wechsler Abbreviated Scale of Intelligence* (Wechsler, 1999)		
L	Vocabulary (verbal IQ)	Provide definitions for spoken words	*r*_*i*_ = .92
Nonverbal	Block Design (performance IQ)	Match a visual pattern using colored blocks	*r*_*i*_ = .88
L	*Woodcock–Johnson III:* Understanding Directions subtest (Woodcock, McGrew, & Mather, 2001)	Obey verbal instructions of increasing complexity	*r*_*i*_ = .83
L	*Expression, Reception and Recall of Narrative Instrument* (ERRNI; Bishop, 2004)	Tell a story from pictures, answer questions about it, and retell it from memory	*r*_*i*_ = .75–.90
	*NEPSY* (Korkman et al., 1998)		
L	Sentence repetition	Repeat sentences of increasing length and complexity	*r*_*i*_ = .82
P	Nonword repetition	Repeat sequences of 2–5 syllables	*r*_*i*_ = .83
P	Oromotor skills	Accurately repeat tongue-twisters	*r*_*MZ*_ = .74
P	Memory for names	Recall name–photograph associations, immediately and after a delay	*r*_*i*_ = .88
N	*Phonological Assessment Battery* (PhAB; Frederickson et al., 1997) Rapid naming	Rapid serial naming of pictures and digits	*r*_*MZ*_ = .71, .68
R	*Test of Word Reading Efficiency* (TOWRE; Torgesen et al., 1999)	Rapidly read real words and nonwords	*r*_*t*_ = .97, .90
A/C	*Neale Analysis of Reading Ability* (NARA-II; Neale, 1997): Stories 1–4	Accuracy, comprehension, and rate for passage reading	*r*_*MZ*_ = .81, .69, .70
A	*Speeded spelling* (unpublished in-house task)	Speeded spelling to dictation in 2 min	*r*_MZ_ = .70

a L = core oral language measure stressing semantic/syntactic skills, used in definition of language impairment (LI); P = tests taxing phonological processes thought to be important for reading; N = tests of rapid serial naming; R = speeded test of word/nonword reading, used in definition of dyslexia (DX); A = measure of accuracy of reading or spelling words; C = measure of reading comprehension.

b *r*_*i*_ = internal consistency reported in test manual; *r*_*t*_ = test–retest reliability reported in test manual; *r*_*MZ*_ = monozygotic twin intraclass correlation in current sample, that is, lower bound estimate of test–retest reliability.

### Standardization of Scores

To compare profiles across language and literacy tests that had been standardized on different populations, all scores on these tests were restandardized relative to a normative set of twins who were selected to be representative of the whole population. This was formed by including all twin pairs who had been in the low-risk subgroup at 6 years of age plus a random subset of the language risk pairs to give a normative group that contained 12% of language risk cases (i.e., reflecting the proportions of high- and low-risk pairs in the whole population). These 98 children had means and standard deviations close to the published normative mean on recently standardized tests (e.g., mean scaled scores relative to published norms on WASI Vocabulary and Block Design mean were 51.4 and 52.1 with *SD*s of 10.6 and 10.2, respectively, compared to expected *M* of 50 and *SD* of 10), confirming that they were comparable to the general population. Their mean and standard deviation raw scores were used to restandardize test scores from the current sample, with a *M* of 100, *SD* of 15, and possible range from 55 to 145. Before carrying out standardization, skewness was calculated, and, for any measure where this differed significantly from zero, a Box–Cox linearity plot (Box & Cox, 1964) was used to identify the optimal transformation to achieve normality. For all measures, transformation gave a distribution of standard scores in which skewness did not differ significantly from zero. Correlations with age were nonsignificant over this restricted range, and so it was disregarded in the standardization.

### Relevant Measures From 4-Year and 6-Year Assessments

Assessments given at 4 and 6 years of age are shown in Table 2 and are described in more detail by Colledge et al. (2002), Kovas et al. (2005), and Bishop et al. (2006). Measures selected for analysis were those that had comparable content to measures given at 9 years (see Table 1). Distributions were inspected for skewness, and Box–Cox transformation was successful in reducing this to nonsignificance except for the phonological awareness measure at 6 years, which showed a ceiling effect.

**Table 2:** *Measures From 4-Year and 6-Year Assessments, With Reliabilities*^a^

Domain	Four-year measure	Six-year measure
Nonverbal ability	Composite of Block Building, Puzzle Solving, Tapping Sequence, and Draw a Design from McCarthy Scales of Children’s Abilities (MSCA; McCarthy, 1972); [*r*_i_ = .89]	Block Design from the Wechsler Abbreviated Scale of Intelligence (WASI; Wechsler, 1999); *r*_i_ = .85
Vocabulary	MCSA Word Knowledge; [*r*_i_ = .89]	WASI Vocabulary; *r*_i_ = .87
Sentence comprehension	Verbal Comprehension subtest, British Ability Scales (Elliott et al., 1996); *r*^a^ = .77	Sentence Structure subtest of the CELF-R; *r*_i_ = .52
Verbal memory	MCSA Verbal Memory for Words and Sentences; [*r*_i_ = .74]	Recalling Sentences from CELF–R (Semel et al., 1987); *r*_i_ = .91
Phonological awareness	Experimental test from Bird, Bishop, and Freeman (1995), 12-item version; *r*^a^ = .76	Experimental test from Bird, Bishop, and Freeman (1995), 18-item version; *r*^a^ = .91
Oromotor skills	Goldman-Fristoe Sounds in Words (Goldman & Fristoe, 1986); *r*^a^ = .96	
Nonword repetition	Children’s Test of Nonword Repetition (Gathercole, Willis, Baddeley, & Emslie, 1994) (20-item version); *r*^a^ = .88	Children’s Test of Nonword Repetition (Gathercole et al., 1994) (full 40-item version); *r*^a^ = .88

a *r*_a_ = coefficient alpha from Twins Early Development Study sample; *r*_i_ = internal consistency from test manual; square brackets denote best estimate from similar composite in test manual.

### Classification of Children According to 9-Year-Old Tests

Children’s reading and language status was coded after first excluding 49 children (13% of sample) whose Block Design scaled scores were more than 1.33 *SD* above or below the mean (i.e., all those included had IQs in range 80–120). The exclusion of children with low nonverbal ability is a standard approach to ensure language or literacy deficits are not simply part of generally low ability. In this study, high-ability children were also excluded to minimize nonverbal differences between the four groups. One further child was excluded because of incomplete data.

#### Dyslexia

We followed the customary procedure of defining dyslexia in terms of accuracy of reading single words and nonwords (Lyon, 2003). To ensure that our definition did not include children whose accuracy was achieved only by very slow reading, we used the timed subtests of the TOWRE (sight word efficiency and phonetic decoding efficiency), taking a cutoff of average score on the two subtests of 83 (i.e., below 13th percentile).

#### LI

LI was coded on the basis of 9-year-old test scores, where the child had a least two scores more than 1.33 *SD* below the normative mean on five core language measures (WASI Vocabulary, WJ understanding directions, ERRNI comprehension, ERRNI MLU, NEPSY Repeating Sentences). This is a similar criterion to that adopted by Catts et al. (2005). See Tomblin, Records, and Zhang (1996)for discussion of this diagnostic approach.

Four groups were formed: 73 children with dyslexia but normal language (DX-only), 35 children with impaired language but normal reading (LI-only), 54 children with both language impairment and dyslexia (LI + DX), and 176 children with neither reading nor language difficulties, referred to as typically developing (TD).

### Analytic Approach

Test scores were compared in one-way analyses of variance (ANOVAs) with the four groups (TD, DX-only, LI-only, and LI + DX) as levels of a group factor and post hoc comparisons conducted using Sidak tests. Mixed model analysis with family as a random effect was used to avoid problems arising from dependencies when two twins from a pair are included in the same analysis (Kenny, Kashy, & Cook, 2006). This adjusts the degrees of freedom in the denominator of the *F* ratio to account for statistical dependency between twins; estimates were made using restricted maximum likelihood in SPSS. Discriminant function analysis was then used to identify the best combination of variables for discriminating the LI-only and LI + DX groups. Finally, for the subset of children who had participated in previous waves of data collection, TD, LI-only and LI + DX groups were compared in terms of test scores obtained at 4 and 6 years of age.

## Results

### Comparison of Mean Test Scores at 9 Years of Age

Table 3 shows mean and standard deviation of standardized scores. Effect sizes (Cohen’s *d*) are also shown for those pairwise comparisons where *p*< .05 after the Sidak correction for multiple comparisons was applied. In relation to the questions outlined in the Introduction, our main interest is in the comparisons between LI-only and TD groups and LI-only and LI + DX groups, but effect sizes are shown for other pairwise comparisons for completeness.

**Table 3:** *Mean* (SD) *Test Scores,* F *Ratios, and Effect Sizes for Pairwise Comparisons on 9-Year-Old Test Battery*

						Effect size (*d*)					
	TD (*n*=176)	DX-only (*n*=73)	LI-only (*n*=35)	LI + DX (*n*=54)	*F* (*df* = 2)	TD vs. DX	TD vs. LI	TD vs. LI + DX	DX vs. LI	DX vs. LI + DX	LI vs. LI + DX
Block design	102.3 (13.09)	98.6 (12.73)	96.6 (11.99)	95.2 (12.65)	5.9 (333.3)	—	—	0.55	—	—	—
Vocabulary^L^	99.2 (13.99)	93.6 (13.21)	81.7 (11.98)	76.9 (13.07)	54.3 (323.4)	0.41	1.35	1.65	0.94	1.27	—
Understand directions^L^	100.7 (13.96)	94.7 (13.54)	82.1 (12.8)	81.9 (13.46)	40.5 (330.6)	0.44	1.39	1.37	0.96	0.95	—
ERRNI story telling	100.9 (14.85)	100.1 (14.52)	91.4 (13.89)	87.6 (14.46)	15.0 (327.8)	—	0.66	0.91	0.61	0.86	—
ERRNI story recall	100.0 (14.83)	100.7 (14.58)	87.9 (14.41)	85.3 (14.55)	20.4 (314.7)	—	0.83	1.00	0.88	1.06	—
ERRNI comprehension^L^	98.4 (14.96)	98.4 (14.58)	90.9 (13.88)	87.6 (14.52)	9.9 (329.6)	—	0.52	0.73	—	0.74	—
ERRNI MLU^L^	101.6 (16.73)	97.1 (16.83)	89.5 (16.13)	84.2 (16.28)	18.4 (306.1)	—	0.74	1.05	—	0.78	—
Repeating sentences^L^	98.7 (14.06)	91.4 (13.22)	83.0 (11.93)	74.4 (13.07)	58.4 (320.6)	0.54	1.21	1.79	0.67	1.29	0.69
Nonword repetition	95.0 (13.79)	88.0 (13.39)	90.6 (12.38)	79.3 (13.33)	21.0 (331.3)	0.52	—	1.16	—	0.65	0.88
Oromotor	96.9 (16.13)	87.1 (15.33)	88.4 (13.96)	73.7 (15.12)	35.3 (324.3)	0.62	0.56	1.48	—	0.88	1.01
Memory for names	101.8 (15.55)	90.2 (14.42)	92.8 (12.89)	85.6 (14.27)	25.7 (311.8)	0.77	0.63	1.09	—	—	—
Rapid name, pictures	100.6 (16.16)	93.0 (15.07)	95.9 (13.65)	85.1 (15.40)	16.1 (318.3)	0.49	—	0.98	—	0.52	0.74
Rapid name, digits	101.9 (14.18)	88.7 (13.32)	97.0 (12.33)	81.2 (13.76)	40.9 (315.5)	0.96	—	1.48	0.65	0.55	1.21
TOWRE words^D^	101.1 (11.73)	75.8 (11.37)	95.6 (10.67)	72.7 (11.30)	142.5 (333.6)	2.19	0.49	2.47	1.80	—	2.08
TOWRE nonwords^D^	101.0 (11.56)	75.7 (11.10)	95.4 (10.27)	72.2 (11.32)	151.2 (329.7)	2.23	0.51	2.52	1.84	—	2.15
NARA accuracy	100.8 (11.71)	75.9 (11.33)	90.6 (10.61)	72.2 (11.26)	140.6 (333.7)	2.16	0.91	2.49	1.34	—	1.68
NARA comprehension	100.2 (11.61)	84.6 (11.31)	87.9 (10.69)	77.0 (11.25)	77.4 (332.8)	1.36	1.10	2.03	—	0.67	0.99
NARA rate	99.4 (14.00)	81.7 (13.08)	91.2 (12.31)	77.8 (13.18)	55.5 (312.4)	1.31	0.62	1.59	0.75	—	1.05
Spelling	100.1 (13.75)	81.5 (13.45)	96.0 (12.98)	79.0 (13.66)	54.7 (328.2)	1.37	—	1.54	1.10	—	1.28

*Note.* Tests denoted L and D were used to define language impairment (LI) and dyslexia (DX), respectively (see text). Supplementary tables showing means and standard deviations for raw scores are available on: http://psyweb.psy.ox.ac.uk/oscci/Miscellaneous.htm. Dash denotes pairwise comparison on Sidak test is nonsignificant.

#### Is reading comprehension disproportionately poor in the LI-only group?

The LI-only group did significantly worse than the TD group on all literacy tests except the spelling test. Nevertheless, the effect size was small for the two TOWRE subtests, with mean scores well within normal limits. On the NARA, there were larger group differences on accuracy, comprehension, and rate, but here too, the means were within the normal range and considerably higher than those obtained by the LI + DX sample. We can conclude that reading comprehension is impaired in the LI-only group, but it is noteworthy that on the NARA they do not show a substantial mismatch between accuracy and comprehension scores, and their text reading fluency, as indexed by the rate measure, is in line with other literacy skills. The overall impression is that these children fare well when required to read or spell single words or nonwords, but do rather more poorly with connected text. However, note that the two TOWRE subtests were used to identify dyslexia and so would be expected to give higher means than the NARA measures because low scorers were explicitly excluded from the LI-only group.

Also noteworthy is the finding that the DX and LI + DX groups do not differ on the literacy measures, except for the NARA comprehension, where the LI + DX do significantly more poorly, consistent with the view that reading comprehension depends on oral language ability.

#### Do LI-only children differ from LI + DX children in severity or profile of oral language skills?

Table 3 shows that the LI-only children score as poorly as the LI + DX children on Vocabulary, Understanding Directions, Memory for Names, and all the narrative indices from ERRNI. The remaining language tests show one of two patterns. The first pattern is where the LI-only group is impaired relative to the TD group but does significantly better than the LI + DX group: This was seen for Repeating Sentences and Oromotor Skills. The second pattern was one where the LI-only group not only did better than the LI + DX group but also was unimpaired relative to the TD group: This was observed for nonword repetition and the two rapid serial naming subtests from the PhAB.

To identify the best combination of variables for distinguishing the LI-only and LI + DX groups, a stepwise discriminant function analysis was carried out, using all variables shown in Table 3 except for the reading and spelling measures. PhAB digit naming was entered at the first step, nonword repetition at the second step, and oromotor skills at the third step. No other variables were significant. This discriminant function predicted group membership correctly for 86.6% of cases; Wilks’s λ = 0.58, χ^2^ = 42.5, *df* = 3, *p*< .001.

#### Reanalysis of rapid serial naming tasks with dyslexia identified on the NARA

The unimpaired performance of the LI-only children on rapid serial naming tasks raised the question as to whether this is a consequence of using a speeded reading test to identify dyslexia. Has this criterion simply selected children who give rapid verbal responses? To address this, children were reclassified using a nonspeeded text reading measure, NARA accuracy, as the criterion for dyslexia (score below 83). This gave fewer LI-only cases (9 children moved from the LI-only to LI + DX category) but it did not alter the pattern of findings on rapid serial naming: These children were still unimpaired relative to the TD group on these measures.

### Comparison of 9-year-old Groups on Data Obtained at 4 and 6 Years

In the Introduction we noted that differences between LI-only and LI + DX groups might reflect *consequences* of literacy skill if performance on language tests was affected by being able to read. It is therefore important to ask whether these two groups differed in cognitive abilities in the preschool years, before they started to read. We were able to address this question with a subset of children who had participated in previous waves of data collection at 4 and 6 years. These children came from the original “language risk” and “low-risk” samples defined on the basis of parental report at 4 years.

Table 4 shows mean scores obtained at 4 years and 6 years for 81 children from the TD group, 17 from the LI-only group, and 29 from the LI + DX group. The DX-only group is excluded; our sampling method meant that most cases of dyslexia were selected for inclusion only at 7 years of age and had not been seen for earlier assessment. As with the 9-year-old data, mixed models analysis was used with family as random effect in order to adjust degrees of freedom to take into account dependencies between twins.

**Table 4:** *Mean* (SD) *Test Scores for 4- and 6-Year-Old Measures,With* t *Tests and Effect Sizes* (d) *for Planned Orthogonal Comparisons*

				TD vs. LI/LI + DX		LI vs. LI + DX	
	TD (*n*=81)	LI-only (*n*=17	LI + DX (*n*=29)	*t* (*df*)	*d*	*t* (*df*)	*d*
4-year-old measures							
Nonverbal ability	97.4 (16.76)	88.9 (16.50)	82.0 (17.06)	3.7 (119.7)	0.71	1.5 (120.5)	—
Vocabulary	96.9 (14.40)	88.2 (5.92)	86.5 (7.58)	3.2 (118.4)	1.02	0.9 (122.0)	—
Sentence comprehension	97.5 (15.38)	82.7 (11.10)	84.3 (13.61)	3.3 (119.9)	1.05	−0.4 (118.0)	—
Verbal memory	96.7 (14.96)	87.7 (11.22)	84.5 (11.20)	3.8 (117.7)	0.85	1.5 (122.0)	—
Phonological awareness	95.7 (15.84)	89.6 (9.48)	87.0 (10.63)	2.5 (110.9)	0.62	1.3 (120.5)	—
Oromotor skills	95.0 (17.06)	81.7 (19.15)	73.9 (12.86)	5.4 (117.7)	1.05	2.5 (116.2)	0.49
Nonword repetition	94.5 (14.71)	86.7 (8.44)	83.0 (9.54)	3.7 (104.0)	0.89	1.3 (118.7)	—
6-year-old measures							
Nonverbal ability	99.3 (13.23)	98.1 (14.46)	90.3 (16.19)	2.8 (105.0)	0.35	1.7 (123.8)	—
Vocabulary	96.9 (14.87)	85.6 (8.44)	77.4 (8.03)	6.6 (118.6)	1.48	3.3 (121.2)	0.99
Sentence comprehension	100.1 (14.01)	89.3 (13.95)	82.3 (10.81)	5.8 (104.8)	1.11	2.0 (123.7)	0.57
Verbal memory	97.5 (16.87)	80.7 (11.98)	72.7 (10.54)	7.1 (121.1)	1.58	3.9 (104.8)	0.71
Phonological awareness	100.0 (14.06)	83.0 (20.36)	85.6 (21.30)	3.4 (93.8)	0.84	−0.1 (116)	—
Nonword repetition	100.6 (14.40)	92.8 (15.21)	87.6 (14.04)	3.7 (96.3)	0.72	1.5 (111.8)	—

*Note.* See Table 2. Dash denotes contrast was nonsignificant.

The tests used to index a given domain are not the same at different ages (see Method section), and this is likely to account for some of the age-related variation. Our interest is therefore not so much in looking at age-related change as in considering whether the LI-only and LI + DX subgroups were distinguishable in the preschool or early school years. Dummy coding of group status was used so that nested planned comparisons could be made, first by dropping the term that distinguished LI-only from LI + DX cases and then by dropping group status from the model altogether. This gave us good power to determine whether LI-only and LI + DX groups differed (in the first comparison), and the second comparison then determined whether these groups were impaired relative to the TD group. Significance was tested by evaluating the chi-square difference in goodness of fit between models.

Although there was a trend for higher means in the LI-only group, in general their mean scores were remarkably similar to those of the LI + DX group at 4 years of age. Although one could argue that poor sensitivity/reliability of tests might play a part in this result, it is noteworthy that the tests demonstrated robust differences between the two LI groups versus the TD group at this age. The one exception was oromotor skills, tested by an articulation test at 4 years. Both LI-only and LI + DX groups were impaired on this test, but the LI + DX group did significantly worse. This speech production task contrasts with the other phonological measures—phonological awareness and nonword repetition—which did not distinguish LI-only and LI + DX groups at 4 years.

A larger gap is apparent between LI-only and LI + DX groups by 6 years of age, though no differences are apparent on the two reading-relevant measures where we might have expected them, that is, phonological awareness and nonword repetition. We considered whether the subset of children who were tested at 4 and 6 years was atypical, but this was not the case. When this same subset of children was compared on 9-year-old measures, the pattern of results was as for the full sample (Table 3), with a marked difference in nonword repetition between LI-only and LI + DX.

## Discussion

Before considering the salient results, two points are worth noting. First, though our analysis relied on categorizing children into discrete groups, this should not be taken to mean that we regard these disorders as qualitatively distinct from normality, or from one another. Definitions of disorder are based on continuous measures, and placement of cutoffs is arbitrary. The main reason for adopting this categorical approach is that it enables us to ask the clinically relevant question of how specific deficits relate to literacy outcomes. The second point to note is that in the past, there were concerns that twins were atypical and at particular risk for language disorders, raising questions about the generalizability of our findings. Contemporary studies, however, suggest that any language delay is mild and most evident in the preschool years (Thorpe, 2006). In general, studies with the TEDS sample have found scores on standardized tests to be close to population norms, and there seems no reason to suppose that the kinds of relations between cognitive skills that were studied here should be different for twins than for single-born children. The good correspondence between our results and those of Catts et al. (2005) gives further confidence on this point.

### Reading Comprehension in Relation to Oral Language Skills

Our study was consistent with the view that oral language skills are more important for reading comprehension than for decoding. Children in the LI-only group had evidence of impaired reading comprehension, despite good decoding skills. Nevertheless, the reading comprehension problems in the LI-only group were not severe, with the mean score just within normal limits. These children also had more problems with reading continuous text than with single-word tests of reading (accuracy and rate) and spelling, although here too, their problems were mild. These mild difficulties could reflect problems in using information from linguistic context and top-down vocabulary knowledge to infer word identities (Nation & Snowling, 2004). Overall, we draw three conclusions: First, as predicted, LI has greater impact on reading comprehension than on single-word recognition or decoding. Second, some children with significant LI are able to achieve literacy skills within normal limits for their age (even when this is assessed using reading comprehension and speed). Third, there were no differences between LI-only and LI + DX groups on the core language measures used to identify LI; thus, these children who read adequately despite LI were not simply the less severely affected. This leads us to consider the profile of abilities of the LI-only group on tests of reading-related skills.

### Phonological Skills and Rapid Serial Naming in Children With LI-Only

Catts et al. (2005) found that children with LI-only were unimpaired on measures of phonological processing. Consistent with their results, we found that nonword repetition was unimpaired in the LI-only group, though these same children had small but significant deficits on measures of oromotor skills and memory for names, tasks that challenge phonological output and phonological memory, respectively.

An intriguing finding was that the LI-only and LI + DX groups did not differ from one another on measures of nonword repetition or phonological awareness at 4 years of age, though their overall performance was worse than that of the TD group. The one measure where they differed at 4 years was articulation. The data suggest that the LI-only group did have some early problems with speech production, but these appear to have been relatively mild, and they did significantly better than the LI + DX group. The lack of differentiation of LI and LI + DX groups on other phonological measures, that is, nonword repetition and phonological awareness in the early years, was unexpected and lends support to the notion that performance on such tasks may be facilitated by orthographic knowledge in those children who develop good literacy. This would be compatible with results of Conti-Ramsden and Durkin (2007), who found that there were reciprocal relationships between nonword repetition skill and reading development in children with SLI.

The most striking finding for the LI-only group was on the rapid serial naming tasks administered at 9 years of age. On these tests, they scored well within normal limits. The discriminant function analysis showed that rapid serial naming was the strongest predictor of LI-only versus LI + DX group status and made an independent contribution from nonword repetition and oromotor skills, which were the only other variables to enter the discriminant function. Contrary to what might have been predicted, rapid serial naming was not related to language level but was a correlate of TOWRE performance. This was most striking for rapid serial naming of digits, where DX-only children fared significantly worse than LI-only children despite the fact that the latter group showed much more severe impairments on most language measures. This finding is consistent with an Italian study that contrasted rapid serial naming in poor readers with and without language delay and found that both groups were impaired at rapid serial naming, not just those with evidence of earlier language problems (Brizzolara et al., 2006). It is unfortunate that we did not include a rapid serial naming measure in prior assessments of children at 4 and 6 years, as this would have helped clarify how far rapid serial naming is influenced by literacy skill (cf. Clarke, Hulme, & Snowling, 2005). Our data suggest it might prove to be a good predictor of risk of early reading difficulties in children with LI, just as it is with typically developing children (Wagner et al., 1997).

There is a parallel here with data from a case report by Groen, Laws, Nation, and Bishop (2006), who studied a child with Down syndrome who had normal scores on the TOWRE despite significant semantic difficulties and low IQ. This child was also given the PHAB rapid serial naming subtests and obtained age appropriate scaled scores (97 and 96, respectively, for Picture Naming and Digit Naming). The data from the LI-only group, coupled with the Groen et al. report, suggest that adequate rapid serial naming skill is associated with good word recognition and decoding, even when oral language skills are impaired.

### Language Skills in Children With DX-Only

Children with DX-only were not the focus of the current article, but our data provide further evidence of subtle oral language difficulties in such children. Note that children with significant language difficulties had been excluded from this group, and they were therefore particularly pure cases of dyslexia. As shown in Table 3, although these children did not meet our criteria for LI and performed well within normal limits on tests of vocabulary, comprehension, and narrative, they nevertheless had mild impairments on nonword repetition, oromotor skills, and memory for names. Although it might be tempting to conclude from this that these mild phonological processing problems are sufficient to impair decoding, we have then to explain why the LI-only group, who did just as poorly on these measures, managed to perform well on the TOWRE subtests, despite having additional oral language deficits. Particularly provocative is the finding that the LI-only children had evidence of phonological difficulties before they started to learn to read, but nevertheless, by 9 years of age they could read words and nonwords rapidly and accurately. Such results challenge causal models that treat early deficits on phonological processing tasks as sufficient to explain reading disability. Previously, it has been noted that children with a combination of deficits in phonological processing and rapid serial naming have more severe problems than those with isolated problems in one of these domains (Morris et al., 1998; Wolf & Bowers, 1999). Our data suggest that for a child with no other oral LIs, reading disability may be apparent only when there is a combination of these two deficits (see also Snowling, 2008).

### Summary of Findings for Children With LI-Only

This study found that most children with language impairments also have reading impairments, but it also confirmed that there is a subgroup of children who have LI but who learn to decode words and nonwords accurately. These children were characterized predominantly by semantic and syntactic problems: They had weak vocabulary, poor sentence comprehension, and poor memory for sentences. Despite these difficulties, they had learned to read single words and spell at an age-appropriate level. Nevertheless, they showed mild deficits in reading connected text, and their comprehension for what they read was rather poor. Although these children had deficits on tests of phonological processing, these were not severe. The most striking feature of children in the LI-only group was their ability to name pictures and digits rapidly, which was well within normal limits. In terms of intervention, it is important to recognize that when a child with LI appears to read accurately, they are likely to have adequate phonological skills, but may not always have good understanding of what they read. This suggests that it would make more sense to focus on training oral language skills, such as vocabulary, rather than phonological processing in such cases.

These data also suggest the intriguing possibility that facility in rapidly naming familiar items somehow protects the child against reading disability. Unfortunately, this appears to be a difficult skill to train (De Jong & Vrielink, 2004), making it hard to apply this knowledge to help children with LI + DX. Nevertheless, other researchers are working toward developing effective interventions that focus on developing reading speed as well as phonological skills (e.g., Wolf et al., 2002), and such an approach would seem to be particularly appropriate for LI + DX children, who typically have deficits in rapid serial naming as well as phonological processing.
